# Effect of expression alteration in flanking genes on phenotypes of *St8sia2*-deficient mice

**DOI:** 10.1038/s41598-019-50006-5

**Published:** 2019-09-20

**Authors:** Keisuke Ikegami, Kazumasa Saigoh, Atsuko Fujioka, Mamoru Nagano, Ken Kitajima, Chihiro Sato, Satoru Masubuchi, Susumu Kusunoki, Yasufumi Shigeyoshi

**Affiliations:** 1 Department of Anatomy and Neurobiology, Kindai University Faculty of Medicine, 377-2 Ohno-Higashi, Osaka-Sayama, Osaka, 589-8511 Japan; 2 Department of Neurology, Kindai University Faculty of Medicine, 377-2 Ohno-Higashi, Osaka-Sayama, Osaka, 589-8511 Japan; 3 Department of Life Science, Faculty of Science and Engineering, Kindai University, 3-4-1, Kowakae, Higashi-Osaka, Osaka, 577-8502 Japan; 4 Laboratory of Animal Cell Function, Bioscience and Biotechnology Center, Nagoya University, Furo-cho, Chikusa-ku, Nagoya, 464-8601 Japan; 5 Department of Physiology, School of Medicine, Aichi Medical University, 1-1 Yazakokarimata, Nagakute, Aichi 480-1195 Japan; 6 Present Address: Department of Physiology, School of Medicine, Aichi Medical University, 1-1 Yazakokarimata, Nagakute, Aichi 480-1195 Japan

**Keywords:** Glycobiology, Neurogenesis, Genetics

## Abstract

ST8 alpha-*N*-acetyl-neuraminide alpha-2,8-sialyltransferase 2 (ST8SIA2) synthesizes polysialic acid (PSA), which is essential for brain development. Although previous studies reported that *St8sia2*-deficient mice that have a mixed 129 and C57BL/6 (B6) genetic background showed mild and variable phenotypes, the reasons for this remain unknown. We hypothesized that this phenotypic difference is caused by diversity in the expression or function of flanking genes of *St8sia2*. A genomic polymorphism and gene expression analysis in the flanking region revealed reduced expression of insulin-like growth factor 1 receptor (*Igf1r*) on the B6 background than on that of the 129 strain. This observation, along with the finding that administration of an IGF1R agonist during pregnancy increased litter size, suggests that the decreased expression of *Igf1r* associated with ST8SIA2 deficiency caused lethality. This study demonstrates the importance of gene expression level in the flanking regions of a targeted null allele having an effect on phenotype.

## Introduction

Glycosylation is one of the important post-translational modifications of proteins. ST8 alpha-*N*-acetyl-neuraminide alpha-2,8-sialyltransferase 2 (ST8SIA2) is one of the enzymes that synthesize polysialic acid (PSA), which is crucial for brain development^1–4^. PSA, a unique linear homopolymer of α2,8-linked sialic acid, is known mainly as a posttranslational modification of the neural cell adhesion molecule (NCAM), a member of the immunoglobulin superfamily^5–7^. Multiple processes, including synaptic plasticity, migration of neural precursor cells, and brain wiring, involve the PSA on NCAM^5–8^. The synthesis of PSA depends on the two Golgi-resident polysialyltransferases, ST8SIA2 and ST8SIA4^1–3,7^. ST8SIA2 is predominantly involved in PSA synthesis during brain development, whereas ST8SIA4 seems to regulate PSA synthesis in the adult brain^6^. Complete abrogation of PSA synthesis in *St8sia2*/*St8sia4* double homozygous null mice causes a postnatal lethal phenotype^2^ with ventricular hydrocephalus^9,10^; however, the knockout of *St8sia2* alone has been reported to show variable developmental phenotypes. One previous report found that *St8sia2*−/− mouse strains are normal^11^, but a more recent one demonstrated that the mutant mice have mild hydrocephalus^12^. The origin of this phenotypic difference has not been delineated.

Gene targeting in mice has helped in delineating the physiological function of many genes in mice. Most of the embryonic stem (ES) cell lines used in gene targeting have been derived from the 129 strain because of its higher efficiency^13,14^. Because many of the resulting mutants show inconsistent behaviour^15,16^, generally, these knockout mice generated from 129 strain ES lines are backcrossed to the C57BL/6 (B6) strain two to seven times before analysis of their phenotypes. However, even after several backcrosses, several centimorgans of flanking region derived from the 129 strain remain linked to the targeted allele, potentially containing hundreds of genes^14^. 129 and B6 strains have many differences between their gene expression profiles, both in the bodies and brains of adults and neonates^17,18^. This phenotypic variation due to flanking gene effects has been reported most often for behavioural phenotypes^19–21^. We therefore must consider the effects of these unreplaced flanking regions, as their presence might mask the pure phenotype of the targeted genes^22^. However, in most cases, the mechanism by which these flanking regions alter the phenotype has not been clarified.

In this study, *St8sia2* null mutant mice with mixed genetic background derived from backcrossing the 129 to B6 have a variable phenotype. We found that the variability in gene expression in the flanking regions of the *St8sia2* locus contributed significantly to these phenotypic differences. By identifying polymorphisms allowing us to map flanking regions, it may allow the explanation of variable mutant phenotypes observed with targeting of a single gene.

## Results

### Flanking regions alter the *St8sia2* knockout phenotype

**Figure 1:**
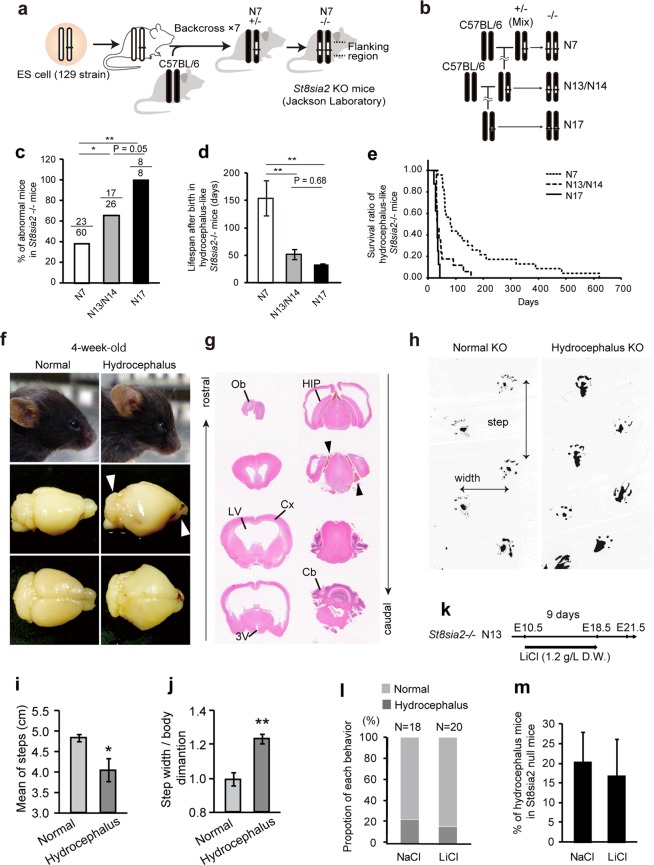
Hydrocephalus is increased by backcrossing ST8SIA2-deficient mice to a C57BL/6 background. (**a**) Generation of *St8sia2*−/− mice. (**b**) Breeding scheme to decrease flanking allele differences in *St8sia2*−/− mice. (**c**) Abnormal null mutant mice as a percentage of total null mutant mice in each generation. *P < 0.05, ***P < 0.001 (Fisher’s exact test). (**d**,**e)** Lifespan (**d**) and survival ratio (**e**) of hydrocephalic null mice. **P < 0.01 (one-way ANOVA, Fisher’s least significant difference (LSD) test, P < 0.01) (**f**) Comparison of lateral views of 4-week-old *St8sia2*−/− mice (upper). Null mice exhibit a bulging forehead (arrow). (**g**) HE-stained coronal sections through identical regions of the brain in severely hydrocephalic animals showing expanded ventricles. (**h**) Gait analysis of 6-week-old female normal (left) and hydrocephalus (right) *St8sia2*−/− mice. (**i**) Short and (**j**) opened gait disorders in hydrocephalic mice. Mean ± SEM, n = 3. *P < 0.05, **P < 0.01, *t*-test. (**k**–**m**) Lithium therapy from E10.5 (through pregnant) by inhibition of GSK3β activity fails to rescue hydrocephalus in *St8sia2*−/− mice.

The mutant mice examined in the present study were backcrossed to B6 only 6 times at the Jackson Laboratory (generation N7, Fig. 1a). A previous study showed that these N7 *St8sia2* homozygous null mutant mice have almost 50% of the NCAM lacking PSA relative to the NCAM of wild-type (WT) controls^1^. We confirmed this observation in N7 using western blotting (WB) (Fig. S1). We further backcrossed to B6 (Fig. 1b) and found an increased ratio of mice exhibiting abnormal development (e.g., developmental delay and hydrocephalus) (Fisher’s exact test, P = 0.145) (Fig. 1c) and significantly decreased lifespan in the adult *St8sia2* homozygous null mutant mice (one-way ANOVA, P < 0.001; Fig. 1d,e). Many null mutant mice survived until 4 weeks of age (Fig. 1e). However, hydrocephalus began to develop in this period (Fig. 1f). This hydrocephalus was severe, with expanded lateral ventricle and third ventricle, deformed hippocampus and habenula, stretched internal capsule fibres, and thinner cortex and corpus callosum (Fig. 1g). We also detected some gait disturbances in homozygous mutant mice including shortened steps, expanded width, and difficulty in walking straight (Fig. 1h–j), as often observed in hydrocephalus patients^23^. Lithium therapy rescues hydrocephalus by inhibition of glycogen synthase kinase 3-β (GSK3β) activity^24^. Lithium therapy from E10.5 through pregnant by drinking LiCl water failed to rescue hydrocephalus in *St8sia2*−/− mice (Fig. 1k–m).  Together, these results demonstrate that enriching the C57BL/6 background region around the *St8sia2* locus may alter phenotypes of *St8sia2*−/− mice.

### Purification of genetic background induced embryonic lethality in *St8sia2*−/− mice

**Figure 2:**
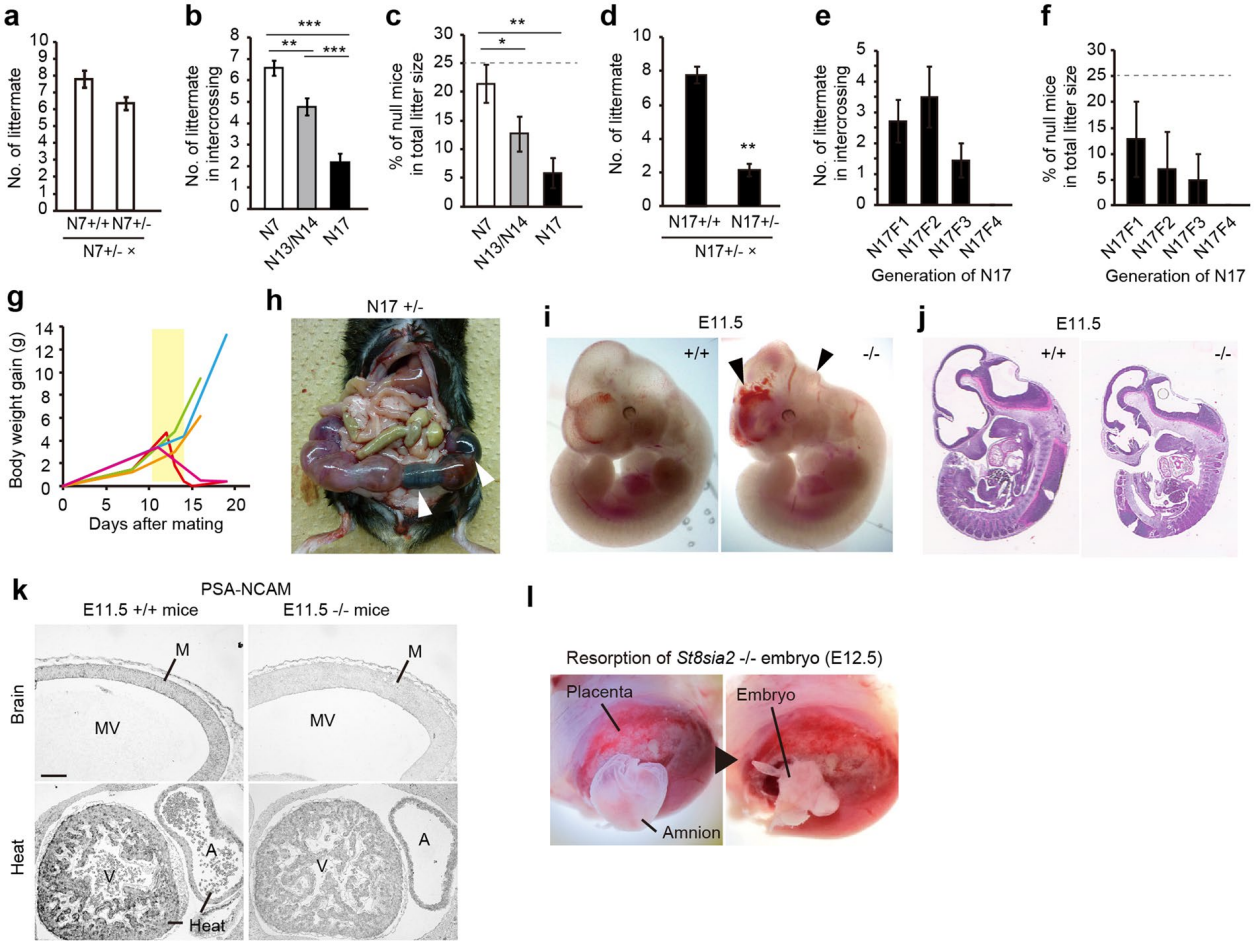
Embryonic lethality induced by additional backcrossing to the C57BL/6 background in ST8SIA2-deficient mice. (**a**) Normal litter size of N7 generation *St8sia2*−/− mice obtained from the Jackson Laboratory. (**b**,**c**) Litter size (**b**) and ratios of null mice (**c**) in each backcross generation. Dotted line in (**c**) indicates calculated percentage of −/− mice. *P < 0.05, **P < 0.01, ***P < 0.001 (one-way ANOVA, Fisher’s LSD test, P < 0.01) (mean ± SEM, n = 20–42). (**d**) Litter numbers in crosses between N17 *St8sia2*+/− mice and +/+ or +/− mice. Mean ± SEM, n = 3–5. **P < 0.01, t-test. (**e**,**f**) Litter size (**e**) and ratios of null mutant mice (**f**) in intercrosses of each generation of N17. One-way ANOVA, Fisher’s LSD test, P > 0.05. Mean ± SEM, n = 5–47. (**g**) Body weight begins to decrease from E10.5 upon intercrossing of +/− female mice. Each line indicates individual data. (**h**) Uterus including dead embryos (white arrowheads) from the intercross of N17 +/− mice. (**i**) Normal (left) and abnormal (right) *St8sia2*−/− embryos (E11.5). Internal bleeding and morphological abnormality were observed (black arrowhead). (**j**) HE-stained sagittal section of +/+ and −/− E11.5 embryos. (**k**) Representative images of PSA-NCAM immunoreactivity in the mesencephalons and hearts of +/+ and −/− E11.5 embryos. Scale bar: 50 μm. M, mesencephalon; MV, mesencephalic ventricle; A, atrium; V, ventricle. (**l**) Resorption of dead null embryo (E12.5).

Intercrossing in N7 heterozygotes yielded normal litter sizes (Fig. 2a). Further backcrossing to the N17 or N18 generation not only increased abnormal development but also markedly decreased litter size and birth rate of homozygous mutant mice (One-way ANOVA, P < 0.01; Fig. 2b,c). Although human sperm is also polysialylated^25^, and lack of PSA inhibited kinesis of the sea urchin sperm and suppressed fertilization^26^, a subset of homozygous mutant mice was sterile (Fig. S2). However, additional backcrosses did not alter the proportion of sterile homozygotes (Fisher’s exact test, P > 0.05; Fig. S2). Litter sizes in crosses between N17 heterozygotes and WT homozygotes also were normal (Fig. 2d), indicating no effect of decreased St8sia2 on sterility. These results suggest that *St8sia2* deletion may induce abnormal gonadal function, which may influence sterility in homozygous mice.

Further backcrossing from N17 decreased litter size (One-way ANOVA, P = 0.14) and birth rates of homozygous mutant mice (One-way ANOVA, P = 0.82; Fig. 2e,f). To estimate the frequency of embryonic lethality, we measured body weight changes in pregnant homozygous mutant mice. We observed that body weight of dams began to decrease from embryonic day 11.5 (E11.5) to E13.5 (Fig. 2g), and dead embryos were observed in homozygous mutant dams’ uteri at E12.5 (Fig. 2h). We also observed that some homozygous mutant embryos displayed internal bleeding and morphological abnormality at E11.5 (Fig. 2i) and were slightly smaller than WT embryos (Fig. 2j). Although PSA-NCAM is expressed in the developing brain^6^, and PSA levels in homozygous mutant mice are lower in the olfactory bulb and cerebral cortex^11^, PSA-NCAM in the brain (Fig. 2k, top) and heart (Fig. 2k, bottom) decreased in our E11.5 homozygous mutant embryos. Interestingly, early death in the *St8sia2*/*St8sia4* double homozygous mutant mice is caused by generalized defects in peripheral organs, including the heart^2^. Dead embryos have been absorbed until E12.5^27^. At E12.5, we observed absorption of dead embryos that did not have a body shape (Fig. 2l), suggesting that this absorption causes body weight reduction, which could be attributed to mid-gestation lethality. Taken together, these data demonstrate that B6-derived flanking regions near the *St8sia2* locus play an important role in hydrocephalus and embryonic lethality.

### C57BL/6-specific gene expression causes early embryonic lethality

**Figure 3:**
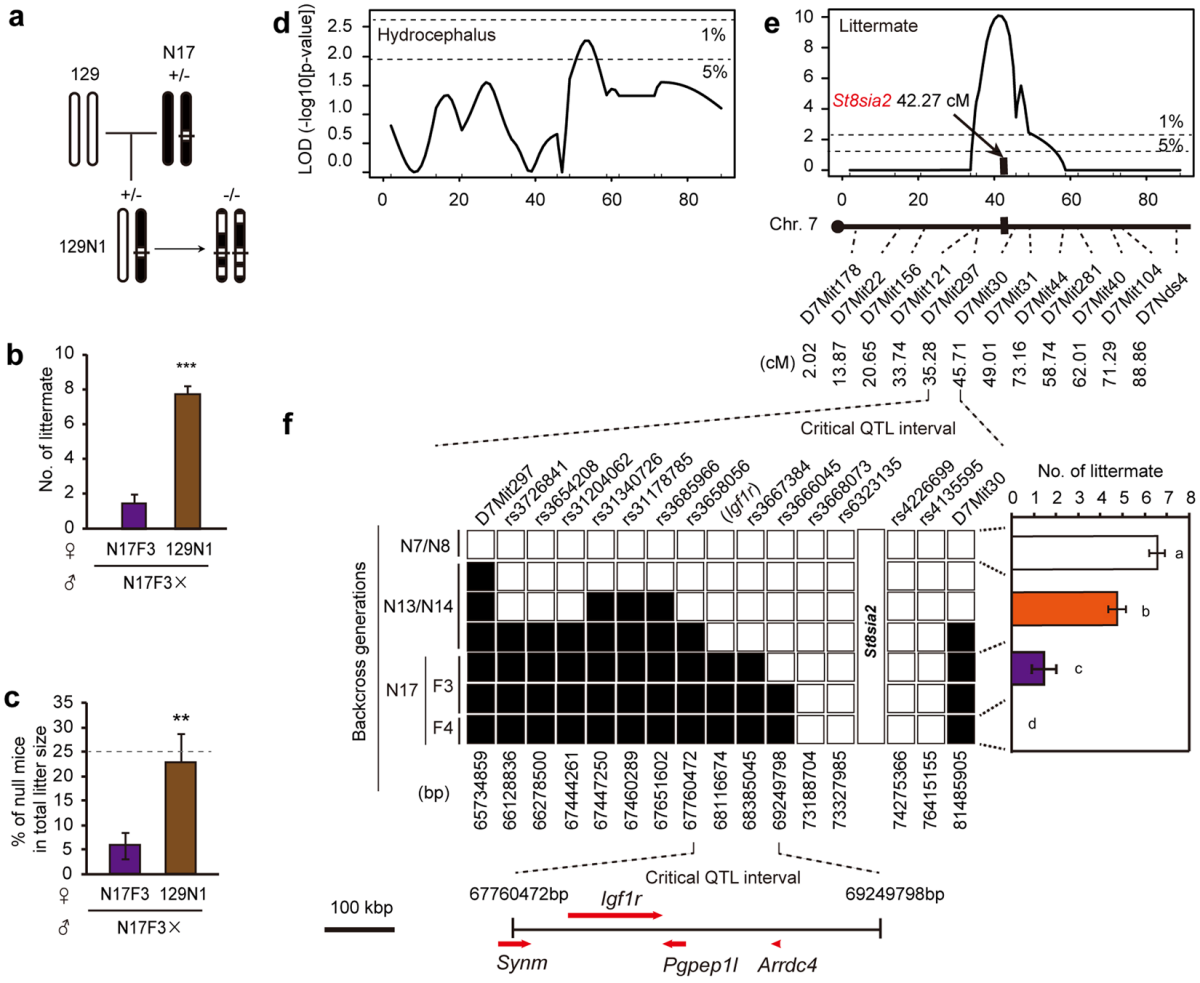
Polymorphism analysis narrowed candidate flanking genes mediating ST8SIA2 deficiency-induced embryonic lethality. (**a**) Breeding scheme to reveal the effect of 129 strain-specific flanking allele on embryonic lethality. N17 mice were backcrossed to 129X1/Sv mice used in the generation of *St8sia2*−/− mice to generate 129N1 mice. (**b**) Litter sizes and (**c**) ratios of null mice in intercross progeny of N17 and 129N1 mice. Mean ± SEM, n = 4–9. **P < 0.01, ***P < 0.001, *t*-test. (**d**,**e)** Logarithm of the odds ratio (LOD score) profiles from the quantitative trait locus (QTL) analysis for hydrocephalus of *St8sia2*−/− mice (**d**) and for littermates by intercross of the same genotypic *St8sia2* hetero mice (**e**) in chromosome 7. The horizontal dashed lines indicate the thresholds for suggestive linkage (5%) and significant linkage (1%). *St8sia2* gene (red) located QTL between D7Mit297 and D7Mit30 for littermates. Microsatellite analysis narrowed the candidate flanking region around *St8sia2* loci. (**f**) Genotypes of single nucleotide polymorphism (SNP) and microsatellite markers on chromosome 7 and litter size in each backcross generations. SNP analysis identified four genes (*Synm*, *Igf1r*, *Pgpep1*l, and *Arrdc4*). Black box: B6 derived allele, White box: heterozygous or homozygous for 129-derived allele. Mean ± SEM, n = 5–47. Means with the same letter are not significantly different (One-way ANOVA, Fisher’s LSD test, P < 0.001).

In targeting *St8sia2*, 129X1/SvJ x 129S1/Sv F1-derived ES cells were used (Jackson Laboratory). To clarify the contribution of the 129/Sv genetic background to the penetrance of the mutant phenotype, we backcrossed N17/N18 males to 129 females (129X1/SvJ) (Fig. 3a). In this mating, litter sizes were normal (Fig. S3a,b). Crossing of the progeny of this backcross with N17F3 heterozygotes restored litter size to a normal level (Figs 3b and S3c), increased the ratio of homozygous births to the expected Mendelian ratio (Fig. 3c), and normalized the development (Fig. S3d) of homozygous mutant mice. This backcross also increased litter size in the crossed homozygous mutant and heterozygous mice (Fig. S3e). To confirm stain specificity, backcrossing to a different inbred strain, Balb/c, had the same rescue effect (Fig. S3a,b). The results are consistent with the hypothesis that the existence of B6 alleles flanking the *St8sia2* locus mediate the penetrance of the *St8sia2*−/− phenotype.

To identify candidate genes related to each phenotype, we performed quantitative trait locus (QTL) mapping analysis for hydrocephalus by intercrossing *St8sia2* heterozygotes using microsatellite markers on chromosome 7 (Fig. 3d,e). This analysis revealed that candidate loci should reside in the interval between markers D7Mit30 to D7Mit44 (significant correlation <5%) (Fig. 3d). The decrease in litter sizes for N17/18 seemed to be caused by recombination between 129 and B6 between D7Mit297 and D7Mit30 (significant correlation <1%) (Fig. 3d,e). To further narrow the flanking region, we carried out single nucleotide polymorphism (SNP) mapping between D7Mit297 and D7Mit30 (Fig. 3f). This analysis identified one flanking region from rs3658056 (67760472 bp) to rs3667384 (68385045 bp). This region includes 4 genes: Synemin [*Synm*], Insulin-like growth factor 1 receptor [*Igf1r*], Pyroglutamyl-peptidase 1-like [*Pgpep1l*], and Arrestin domain containing 4 [*Arrdc4*] (UCSC Genome Browser). Taken together, these data suggest that one of these four genes may be responsible.

### Flanking gene *Igf1r* induces embryonic lethality in *St8sia2*−/− mice

**Figure 4:**
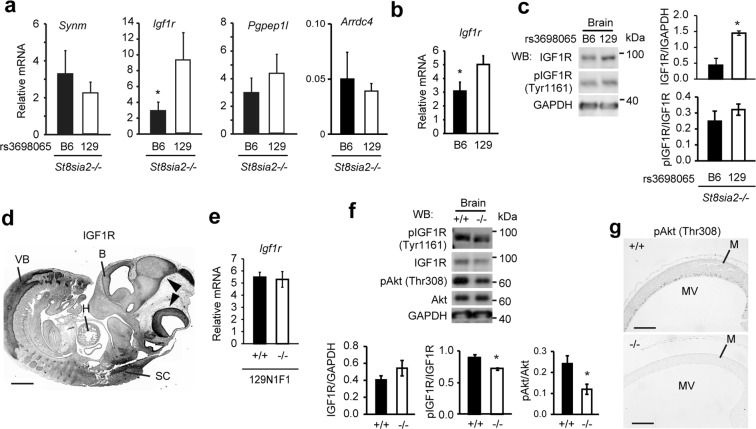
B6-dependent *Igf1r* suppression modulates embryonic lethality in *St8sia2* homozygous null mutant mice. (**a**) Strain-dependent expression of flanking genes *Synm*, *Igf1r*, *Pgpep1l*, and *Arrdc4* in embryonic head at E10.5 on B6 and 129 backgrounds at rs3698065. Mean ± SEM, n = 3. *P < 0.05, *t*-test. (**b**) Expression of *Igf1r* in embryonic head of B6 and 129 mice. Mean ± SEM, n = 11–15. *P < 0.05, *t*-test. (**c**) IGF1R expression and phosphorylation of IGF1R (Tyr1161) in embryonic head at E10.5 on B6 and 129 backgrounds at rs3698065. Mean ± SEM, n = 3. *P < 0.05, *t*-test. (**d**) Immunohistochemical IGF1R localization in WT embryos (E11.5). Scale bar: 500 μm. B, brain; VB, vertebral body; H, heart; SC, spinal cord. (**e**) Expression of *Igf1r* in embryonic head of *St8sia2*−/− mice. (**f**) Effect of *St8sia2* deletion on phosphorylation of IGF1R (Tyr1161) and Akt (Thr308) by WB analysis. *St8sia2* deletion does not affect IGF1R level, but increase activation of IGF1R and Akt. Mean ± SEM, n = 3. *P < 0.05, *t*-test. (**g**) Representative images of phosphorylated Akt (Thr308) immunoreactivity in the brains of homozygous WT and homozygous mutant E11.5 embryos. Scale bar: 100 μm. M, mesencephalon; MV, mesencephalic ventricle.

We analysed the expression of the four candidate genes at E11.5 in N17 homozygous mutant embryos with both the B6 and 129 alleles at rs3698065 (Fig. 4a). Those with the B6 allele had low *Igf1r* expression levels in the head (Fig. 4a), consistent with *Igf1r* expression in embryos of B6 and 129 strain (Fig. 4b) and previous studies using B6 and 129^17,18^. In addition, quantification of the expression of the other genes between D7Mit297 and D7Mit30 did not show any significant differences (Fig. S4). We also found decreased IGF1R, but no alteration in phosphorylation of IGF1R in B6-enriched embryonic head (Figs 4c and S5a), indicating that the B6 genetic background on *Igf1r* gene appears to supress its expression.

IGF1 is primarily secreted from the liver as a result of stimulation by growth hormone (GH)^28^. IGF1 and IGF1R are very important for normal growth and development. *Igf1r* null mutant mice have perinatal mortality, with impaired development of the diaphragm and intercostal muscles^29^. Our immunohistochemistry demonstrated IGF1R expression in the whole body, especially in the brain and vertebrae, of E11.5 embryos (Fig. 4d), consistent with a previous report^29^. Interestingly, IGF1R activation is known to be suppressed by desialylation in skeletal myoblasts^30^; indeed, although *Igf1r* expression and IGF1R levels showed no change in *St8sia2* null mutant mice (Figs 4e,f and S5b), IGF1R phosphorylation was slightly but significantly decreased (Figs 4f and S5b). The IGF/GH axis has been shown to mediate the PI3K/Akt pathway^31^ and play roles in the promotion of cell proliferation and the inhibition of cell death^32^. ST8SIA2 and ST8SIA4 also are involved in development via the PI3K/Akt pathway in humans^33^. To clarify the effects of the *St8sia2* deletion on PI3K/Akt signalling, we quantitated the amount of phosphorylation of Akt, and found its decrease in 129-enriched *St8sia2*−/− embryos (Fig. 4f,g). This suggests that ST8SIA2 modulates the activation of the IGF1R and PI3K/Akt pathways through PSA-NCAM, and that sufficient amounts of IGF1R are necessary for normal development in the absence of *St8sia2*.

### Administration of IGF1 rescues embryonic lethality in *St8sia2* null mutant mice with low IGF1R

**Figure 5:**
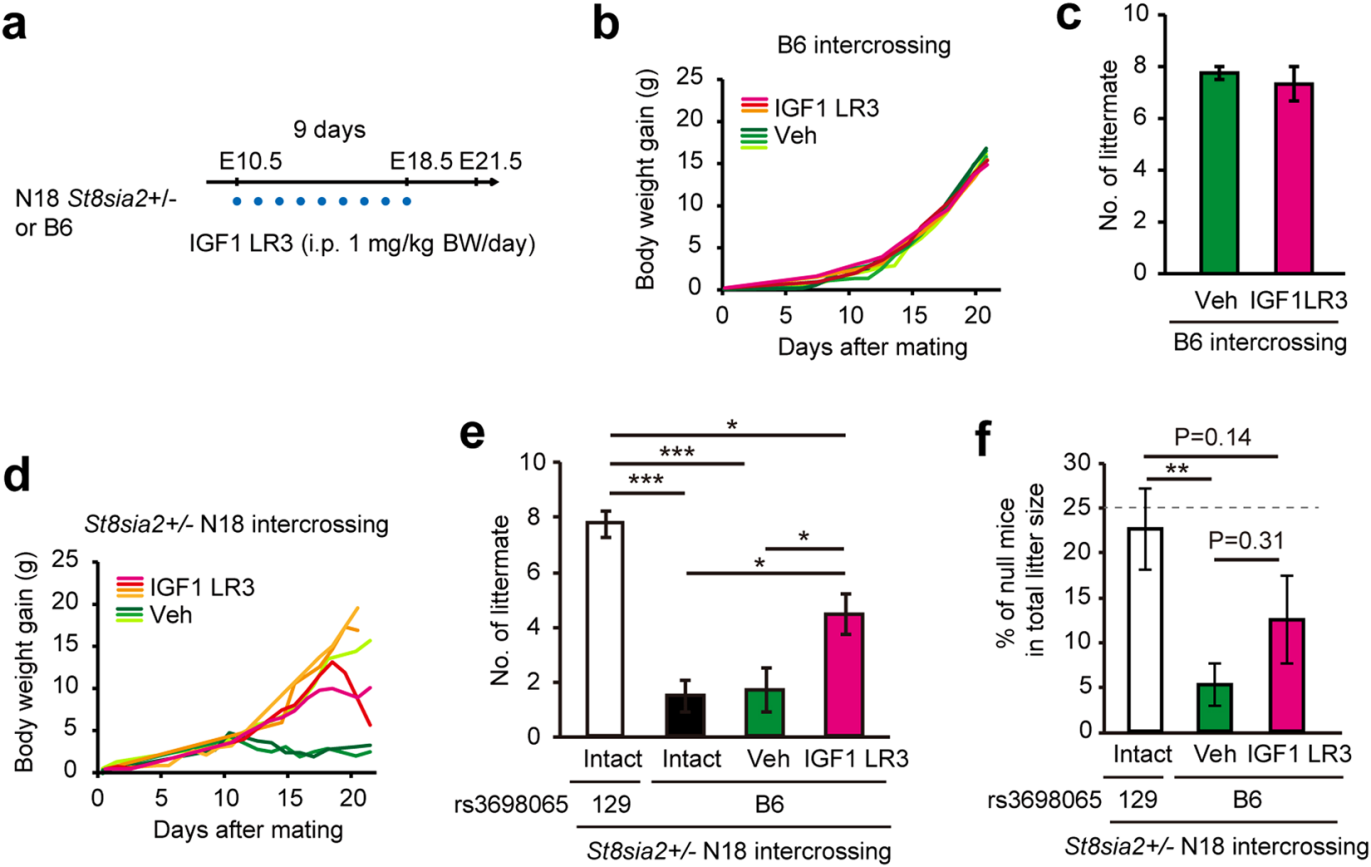
IGF1 analogue administration increases litter size. (**a**) Scheme for IGF1 LR3 injection. IGF1 LR3 was intraperitoneally (i.p.) administered from E10.5 for 9 days to pregnant B6 and N18 *St8sia2*+/− female mice. Blue dots indicate the time points of injections. (**b**) Maternal body weight gain as a function of days after mating in B6. Each line indicates individual data. (**c**) IGF1 LR3 has no effect on litter size. (**d**) Maternal body weight gain as a function of days after mating in N18 *St8sia2*+/− intercrossing. Each line indicates individual data. (**e**) Effect of IGF1 LR3 administration on litter size in intercrosses of B6-background *St8sia2*+/− mice. Mean ± SEM, n = 6–9. *P < 0.05, **P < 0.01, ***P < 0.001 (One-way ANOVA, Fisher’s LSD test, P < 0.001). (**f**) Effect of IGF1 analogue injection on the ratio of *St8sia2*−/− mice in live births. Mean ± SEM, n = 6–9. **P < 0.01 (One-way ANOVA, Fisher’s LSD test, P < 0.01).

To verify this hypothesis, we administered IGF1 analogue (IGF1 LR3) injections daily to pregnant B6 and N18 heterozygotes (Fig. 5a). In wild-type B6 females, IGF1 LR3 injection did not result in any difference in body weight changes (Fig. 5b) and litter sizes (Fig. 5c), indicating there was no effect on normal mothers and embryos. On the other hand, in *St8sia2* mutant mice, it suppressed body weight reduction or delayed its timing during pregnancy, compared with that in the control group (Fig. 5d). Moreover, IGF1 LR3 partially restored litter sizes from those in N13/N14 generations (Fig. 5e), indicating the existence of other responsible factors. IGF1 LR3 did not significantly increase the ratio of null mutant mice in relative to with vehicle treated group, but there was an insignificant increase (Fig. 5f). These results suggest that ST8SIA2 deficiency may cause embryonic lethality through B6-allele dependent expression and ST8SIA2-dependent activation of IGF1R. However, it was possible that the condition of the mother affected the embryonic lethality. Mainly there were two possibilities: (1) Embryonic lethality was due to a reduction of maternal PSA or suppression of IGF1R activation due to maternal heterozygous *St8sia2*, (2) Suppression of *Igf1r* expression due to the maternal B6-allele may affect embryonic lethality. To examine these possibilities, we next transferred *in vitro* fertilization (IVF) embryos from a heterozygous intercrossing of 129N1F1 and N18 mice to wild-type ICR female mice. As a result, the number of littermates decreased (Fig. S6a), and no deficient mice were born in N18 mice (Fig. S6b). This suggests that embryonic lethality was not affected by maternal genotype and genetic background. Thus, IGF1 LR3 is likely to act directly on the foetus. However, it is unclear whether the IGF1 LR3 analogue crosses the placenta. Since the maternal IGF1 level can affect foetal circulating IGF1 levels in humans without passing through the placenta^34^, it is possible that injected IGF1 LR3 acted on the foetal placenta without passing through the placenta and indirectly raised foetal IGF1. This will be addressed in future studies.

## Discussion

In this study, we observed that *St8sia2* homozygous mutants increased incidence of hydrocephalus and embryonic lethality after their genetic background was purified by additional backcrosses to B6 up to almost inbred mice level (Figs 1 and 2). These results are intriguing because there are few reports that demonstrate phenotypic changes due to the alleles flanking targeted genes. Recent CRISPR/Cas9 systems for modifying genes make it possible to produce knockout animals with a uniform genetic background because of using B6 fertilized oocytes or ES cells. However, knockout mice created with conventional methods using 129 strain ES cells can exhibit flanking gene effects that dramatically affect phenotypic changes in null mutants^19–21^. Previous studies showed that the deletion of *St8sia2* did not have dramatic phenotypic changes and showed variable phenotypes^11,12^. In this study, we showed that the B6 genetic background of *St8sia2* homozygotes mice resulted in higher mortality rate in midgestation (Fig. 2). It is still possible that the genetic background of the original line used to construct mutant mice contributes to phenotypic variation.

In the *St8sia2* homozygous mutant mice with B6 background, both deficiency of ST8SIA2 and decreased expression of IGF1R seemed to contribute to abnormal development (Fig. 4). IGF1R and ST8SIA2 were expressed in the embryonic brain of wild type mice (Figs 2 and 4), which was consistent with data from the EMAGE database. IGF1R has been reported to be essential for normal development^29,35^. ST8SIA2 is involved in PSA biosynthesis on the neural cell adhesion molecule (NCAM)^6^ and the resulting product, PSA-NCAM, plays an important role in the migration of neurons^4,36^. Therefore, it is possible that the two conditions worked synergistically and resulted in the abnormalities being more severe. Furthermore, the interaction between the ST8SIA2 and IGF1R might contribute to the construction of the pathological state. In this study, *St8sia2* deletion did not alter IGF1R level, but decreased PSA-NCAM level (Figs 2 and S1) and suppressed activation of IGF1R and its signal cascade (Fig. 4). This is consistent with reports that PSA-NCAM enhances IGF1R activity^35^. Appropriate interaction of ST8SIA2 and IGF1 may be necessary for normal development. B6-enriched *St8sia2* homozygous mutant mice showed decreased expression of both *Igf1r* mRNA and IGF1R protein (Fig. 4); this observation is consistent with previous studies on neonate and adult brain of B6 and 129^17,18^.

Differential analysis of SNP variations around the *Igf1r* locus specific to B6 mice, in comparison with those of 129 and Balb/c mice, revealed several B6-specific SNPs only inside the introns, mainly intron 2, but not in exons or the promoter region (Fig. S7). It is possible that this intron is the cause of the decreased expression. Previous studies suggest two possibilities. The first is the methylation of introns. Recent study has identified a novel differentially methylation in the largest intron of human *IGF1R* gene^37^. DNA hypermethylation was reported in this intron in the mammary gland of parous mice^32^. This intron also allows other epigenetic modifications, including methylation of lysine 4 on histone 3 (UCSC Genome Browser)^32^. Therefore, it is possible that genetic variation of this region was involved in *Igf1r* function in B6 mice. One other possibility is the effect of intron retention. *Igf1r* gene has intron-retained splicing variants and isoforms (Ensemble asia), resulting in degradation of the mRNA by a monitoring system called nonsense-mediated decay (NMD)^38^. In mammals, this mechanism can downregulate up to 35% of alternatively spliced transcripts^39^. Therefore, intrinsic SNP may affect *Igf1r* expression through these modification on the introns.

In the present study, we showed that the higher number of backcrosses to B6 increased the ratio of hydrocephalus in *St8sia2* homozygous mutant mice (Fig. 1). B6 mice tend to be more hydrocephalic relative to other inbred strains^40^. Although we found a weak but suggestive QTL region for hydrocephalus 3’ of *St8sia2* on Chromosome 7 (Fig. 3), how hydrocephalus occurs remains to be elucidated. We observed that hydrocephalus in mutant mice with the more B6 background was severe, with drastic enlargement of lateral ventricles (Fig. 1). As we could not observe the enlarged fourth ventricle (Fig. 1), closure may occur in the aqueduct of the midbrain. Although the cerebrospinal fluid is absorbed from capillaries distributed over the brain, especially ependymal cells and choroid plexus^9^, morphological abnormality could not be observed (Fig. 1). In both lethal embryos and hydrocephalic adults, we observed internal bleeding in the brain, specifically in the dorsal choroid plexus and in cerebrospinal fluid (Figs 1 and 2). Intraventricular and subarachnoid haemorrhage can cause hydrocephalus. Furthermore, the surfaces of endothelial cells in blood vessel have been reported to be highly sialylated^41^. We hypothesize that the absence of ST8SIA2 may lead to capillary abnormalities, leading to hydrocephalus and embryonic lethality through internal bleeding.

In summary, we have shown that genes flanking *St8sia2* regulate the phenotypes of *St8sia2* homozygous null mutants. The potential for data misinterpretation due to such effects has caused many problems^19–21^. This report clearly demonstrates that flanking genes affect phenotypes in mice homozygous for the targeted null allele. Confirmation of such effects may make it possible to reveal masked gene functions in mice homozygous for other targeted null alleles.

## Methods Summary

### Animals and drug treatment

Mice were maintained at a temperature of 23 ± 1 °C. Food (CE-2; CLEA) and water were provided *ad libitum*. All animals were housed in plastic cages under a 12-h light, 12-h dark (L/D) cycle (light on 0700 h). Individual cage illumination was achieved by using LEDs (NSPW500BS, Nichia, Japan) through frosted glass. All animal experiments were approved by the Committee of Animal Care and Use of Kindai University School of Medicine, and all experimental procedures were conducted in accordance with institutional guidelines for the use of experimental animals.

*St8sia2* subcongenic mice (B6.129-*St8sia*2^tm1Jxm^/J) backcrossed to C57BL/6J for 6 generations were obtained from Jackson Laboratory (Bar Harbor, ME, USA) and defined as the N7 generation. We further backcrossed these N7+/− mice with C57BL/6J (CLEA, Tokyo, Japan) mice for 6 or 7 to 10 or 11 generations to generate N13 or N14 generations, showing hydrocephalus and embryonic lethality at N17 and N18 respectively. For rescue experiments, N17 or N18 generation mice were crossed with 129 (129X1/SvJJmsSlc; Nihon SLC, Shizuoka, Japan) and Balb/c (BALB/cCrSIc; Nihon SLC, Shizuoka, Japan) mice. We defined their pups as 129N1 and BalbN1, respectively. After intercrossing the strains mentioned above with *St8sia2* heterozygotes, subsequent generations were defined as F1, F2, F3, and F4.

Following timed matings, embryos were isolated at embryonic day 11.5 (E11.5) and fixed in 4% paraformaldehyde in PBS for 12–20 h. As treatment for hydrocephalus during N13 *St8sia2*+/− intercrossing, pregnant mice were administered 1.2 g/L D.W. lithium chloride (Nakarai Tesque, Kyoto, Japan) in their drinking water from E10.5 (through pregnant) as previously reported^24^, and then, following birth, we genotyped and counted numbers of mice suffering from hydrocephalus. For rescue of embryonic lethality by N18 *St8sia2*+/− and B6 intercrossing, 1 mg/kg body weight of an IGF1 analogue (IGF1 LR3) (946870-92-4, Purity Peptide Labs) was injected i.p. from E10.5 to E18.5 into pregnant B6 and N18+/− mice^42^; then, after birth, we determined litter sizes. For WB analysis of PSA, brains were collected from 10-week-old male *St8sia2* +/+ and −/− mice kept under LD conditions. For WB analysis of IGF1R and activated IGF1R, embryonic heads were collected from N17 *St8sia2*−/− mice on B6 and 129 backgrounds at *Igf1r* SNP (rs3698065) at E10.5, and from 129N1F1 *St8sia2* +/+ and −/− mice at E11.5 respectively.

### Genotyping

All mice were genotyped before and after behavioural testing by polymerase chain reaction (PCR). Tail DNA was extracted using the GenScript TissueDirect Multiplex PCR System (GenScript Corporation, Piscataway, NJ, USA) and subjected to PCR using a buffer containing 1.0 U/50 μL Taq DNA polymerase, 45 mM KCl, 2.5 mM Mg^2+^, 200 μM dNTP (Eppendorf Hotmastermix, Eppendorf AG, Hamburg, Germany) and 0.4 μM of the corresponding primers (forward primers for WT and *St8sia2*−/− were 5′-cctctctcgtgtacccactgccat-3′ and 5′-aggctccctcactgctgtcta-3′, respectively, and the shared reverse primer was 5′-gggaacagcgctcataagat-3′). The PCR conditions were 95 °C for 10 min, followed by 35 cycles at 94 °C for 30 s, 63 °C for 1 min, and 72 °C for 1 min, with a final extension of 72 °C for 7 min. PCR products were resolved on a 2% agarose gel in Tris-Acetate-EDTA Buffer (TAE) to identify the diagnostic bands (700 bp for WT and 380 bp for KO).

### Behaviour test

Gait analyses were performed in six-week-old female normal and hydrocephalic *St8sia2*−/− mice by painting hind limbs with India ink. We made the mice walk on the clear film, and then measured length and width of their steps and body width.

### QTL analysis

The entire group of 36 male N17 generation *St8sia2*−/− animals for hydrocephalus and 43 couples of the same generation *St8sia2*+/− mice as controls were genotyped using 11 microsatellite markers in chromosome 7. A standard genome scan was conducted using R/qtl^43^ to detect loci associated with hydrocephalus and litter size. For each analysis, genome-wide thresholds for suggestive (P < 0.05) and significant (P < 0.01) QTL were determined by 1000 permutations. Transformation of the data allows for permutation-based thresholds that are the same for all traits.

### Polymorphism analysis

DNA was extracted from the tail by ethanol extraction after proteinase K digestion as previously described^44^. In total, 14 microsatellite markers of mice chosen from the Mouse Microsatellite Data Base of Japan (MMDBJ) (http://shigen.nig.ac.jp/mouse/mmdbj/top.jsp) and ENSEMBL genome web browser (http://asia.ensembl.org/Mus_musculus/Marker) were tested by amplification of genomic DNA from C57BL/6J and 129X1/Sv mice (Supplementary Table S1). The PCR conditions for using Ex Taq (Takara) were 95 °C for 2 min, followed by 40 cycles of 94 °C for 30 s, 55 °C for 30 s, and 72 °C for 1 min, with a final extension at 72 °C for 4 min. PCR products were resolved on a 2–4% agarose gel in TAE.

For SNP analysis, we chose markers within a restricted chromosome 7 region based on data from Mouse Genome Informatics (MGI; http://www.informatics.jax.org/snp) and Mouse Genomes Project - Query SNPs (Wellcome Trust Sanger Institute). In a comparative analysis of *Igf1r* gene between mouse strains, the 10 kbp upstream and downstream region of a gene was used. Primer sequences are described in Supplementary Table S2. The PCR conditions were 95 °C for 2 min, followed by 28 cycles of 94 °C for 30 s, 55 °C for 30 s, and 72 °C for 30 s, with a final extension at 72 °C for 4 min. PCR products were resolved on a 2–4% agarose gel in TAE to identify the genotypes. To detect B6-specific flanking region, we defined 129 homo and hetero as the same genotype (white).

### Quantitative PCR

Total RNA was isolated from embryonic heads using Sepazol (Nakarai Tesque, Kyoto, Japan). After genomic DNA removal by DNase I treatment (Sigma), a 0.5 μg aliquot of each total RNA preparation was reverse transcribed using ReverTra Ace (Toyobo) and 2.5 μM oligo (dT). Real-time PCR (qPCR) was performed with an ABI PRISM 7900HT, in a total volume of 10 μL using SYBR Premix Ex Taq II (Tli RNaseH Plus) (Takara, Shiga, Japan), according to the supplier’s instructions. mRNA quantification was performed with two primers as described in Table S3. The resulting threshold cycle (Ct) values from the cDNA amplifications were normalized to the Ct values for 18S rRNA.

### Protein extraction

Embryonic heads and adult brains (for PSA-NCAM analysis, dentate gyrus of the hippocampus) were homogenized using a pestle and sonicated with PBST (0.02 M PBS, 0.1% Triton-X-100, pH 7.4) containing 1% protease inhibitor cocktail (Sigma-Aldrich) as previously described^45^. After 10 min of incubation on ice, lysates were centrifuged at 10,000 × *g*, at 4 °C for 20 min, and the supernatant was collected. Protein concentration of the supernatant was measured using the Micro BCA protein assay reagent (Thermo Fisher Scientific), and the supernatant was stored at −80 °C.

### Histological analysis

Paraffin sections (5 µm) of PFA fixed embryos or hydrocephalus brains were used for haematoxylin and eosin (HE) staining and immunohistochemistry^46^. Immunohistochemistry to detect *Igf1r* was performed using a rabbit polyclonal antibody to IGF1R (1:100; sc-713, Santa Cruz Biotechnology), a monoclonal antibody to phospho-Akt (Thr308) (1:1000; 9275, Cell Signalling Technology), and a mouse monoclonal IgM to PSA-NCAM (2-2B) (1:100; MAB5234, Millipore), as previously reported^45^. For PSA-NCAM, peroxidase-conjugated goat polyclonal antibody against mouse IgM (1:500; Kirkegaard & Perry Laboratories) was used as secondary antibody.

### WB analysis

WB was performed as previously reported^45^. Membranes were incubated with the following primary antibodies: rabbit polyclonal antibodies against NCAM (1:2,500) (Chemicon International), IGF1R (1:200; Santa Cruz Biotechnology), and phosphor- IGF1R (Tyr1161) (1:100; sc-101703, Santa Cruz Biotechnology), rabbit monoclonal antibodies against phospho-Akt (Thr308) (1:1000; 9275, Cell Signalling Technology), and Akt (1:1000; 4685, Cell Signalling Technology), and mouse monoclonal antibodies against polysialic acid (PSA)-NCAM (1:2000; Millipore) and GAPDH (1:2000; Affinity Bioreagents); HRP-conjugated goat polyclonal antibody against rabbit and mouse IgG (1:10,000; Cell Signalling Technology) or IgM (1:10,000; Kirkegaard & Perry Laboratories).

### *in vitro* fertilization (IVF)

The IVF was commissioned to the Animal Collaborative Center at Kindai University Faculty of Medicine. Briefly, female heterozygotes (3–4 weeks old) were super ovulated by i.p. administration of CARD HyperOva (0.1 mL, Kudo, Japan) followed 48 hr later by 7.5 IU (i.p.) human chorionic gonadotrophin (hCG). About 20 hr after hCG injection, oocytes were collected from the oviduct, and then were co-incubated with gametes for 5 hr at 38.5 °C in an atmosphere of 5% CO_2_. The fertilized eggs were then transferred to KSOM medium (Merck Millipore), cultivated overnight in a 5% CO_2_ incubator at 37 °C, and 50 two-cell stage embryos were then transferred into the oviducts of pseudopregnant ICR females at 0.5 d.p.c. (Nihon CLEA).

### Statistical analyses

All data are presented as mean ± SEM. Statistical comparisons were made using Excel-Toukei 2012 software (Social Survey Research Information Co. Ltd., Osaka, Japan). Student’s t-test was used for comparison of two groups and one-way ANOVAs with Fisher’s least significant difference (LSD) tests when more than two groups were compared. Ratios were statistically analysed by Fisher’s exact test. Differences with *p* < 0.05 were considered statistically significant.

## Supplementary information

Supplementary information
