# Supplementary material for: Effect of expression alteration in flanking genes on phenotypes of St8sia2-deficient mice
Source: Sci Rep. 2019 Sep 20;9:13634. doi: 10.1038/s41598-019-50006-5 (PMC6754417; doi:10.1038/s41598-019-50006-5)
Supplement: Supplementary file 1 — Supplementary information [file 41598_2019_50006_MOESM1_ESM.pdf]

Supplementary Information

Effect of expression alteration in flanking genes on phenotypes of *St8sia2*-deficient mice

Keisuke Ikegami [1,#*], Kazumasa Saigoh [2,3], Atsuko Fujioka[1], Mamoru Nagano[1], Ken Kitajima [4], Chihiro Sato[4], Satoru Masubuchi[5], Susumu Kusunoki [2], Yasufumi Shigeyoshi[1*]

[1] *Department of Anatomy and Neurobiology, Kindai University Faculty of Medicine, 377-2 Ohno-Higashi, Osaka-Sayama, Osaka 589-8511, Japan*

[2]*Department of Neurology, Kindai University Faculty of Medicine, 377-2 Ohno-Higashi, Osaka-Sayama, Osaka 589-8511, Japan*

[3]*Department of Life Science, Faculty of Science and Engineering, Kindai University, 3-4-1, Kowakae, Higashi-Osaka, Osaka, Japan, 577-8502, Japan*

[4] *Laboratory of Animal Cell Function, Bioscience and Biotechnology Center, Nagoya University, Furo-cho, Chikusa-ku, Nagoya 464-8601, Japan,*

[5]*Department of Physiology, School of Medicine, Aichi Medical University, 1-1 Yazakokarimata, Nagakute, Aichi, 480-1195, Japan*

[#]*Present address: Department of Physiology, School of Medicine, Aichi Medical University, 1-1 Yazakokarimata, Nagakute, Aichi, 480-1195, Japan*

[*] To whom correspondence should be addressed.

Keisuke Ikegami, Ph.D.

Assistant Professor of Department of Physiology, School of Medicine,

Aichi Medical University,

1-1 Yazakokarimata, Nagakute, Aichi, 480-1195, Japan

E-mail: ikegami.keisuke.910@mail.aichi-med-u.ac.jp

Phone +81-561-62-3311; Fax +81-561-63-1289

Yasufumi Shigeyoshi, M.D. and Ph.D.

Professor of Department of Anatomy and Neurobiology,

Kindai University Faculty of Medicine,

377-2 Ohno-Higashi, Osaka-Sayama, Osaka 589-8511, Japan

E-mail: shigey@med.kindai.ac.jp

Phone/Fax +81-72-368-1031

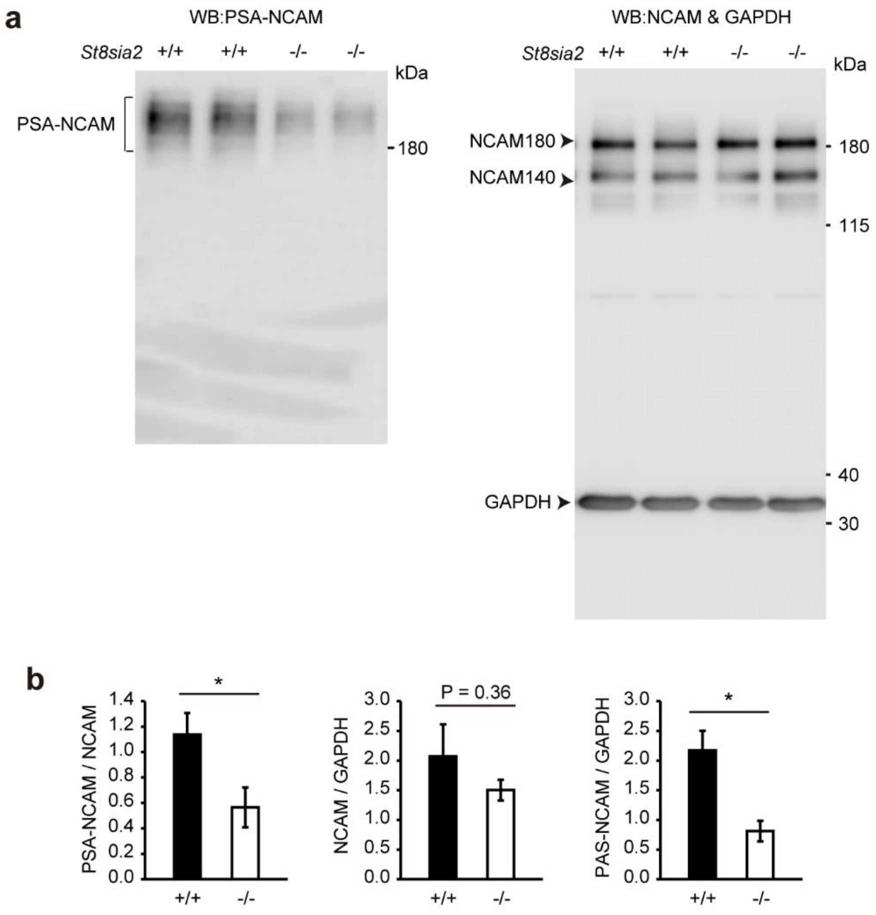

Supplementary figure S1: Polysialic acid in NCAM in *St8sia2* -/- brains

a, Western blotting and of polysialic acid (PSA) in NCAM (PSA-NCAM; bracket), total NCAM (arrowhead), and GAPDH in the dentate gyrus of the *St8sia2* +/+ and -/- hippocampus. **b,** Quantification analysis of relative PSA-NCAM and NCAM. Relative PSA-NCAM decreased in the *St8sia2* -/- hippocampus. Mean ± SEM, n = 4. *P < 0.05, *t*-test.

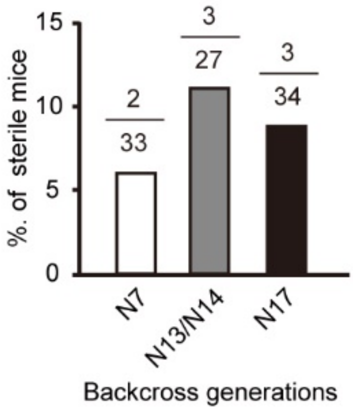

Supplementary figure S2: Ratio of sterile mice for backcross generations

The proportion of sterile mice in each generation was not statistically different (Fisher's exact test, P>0.05).

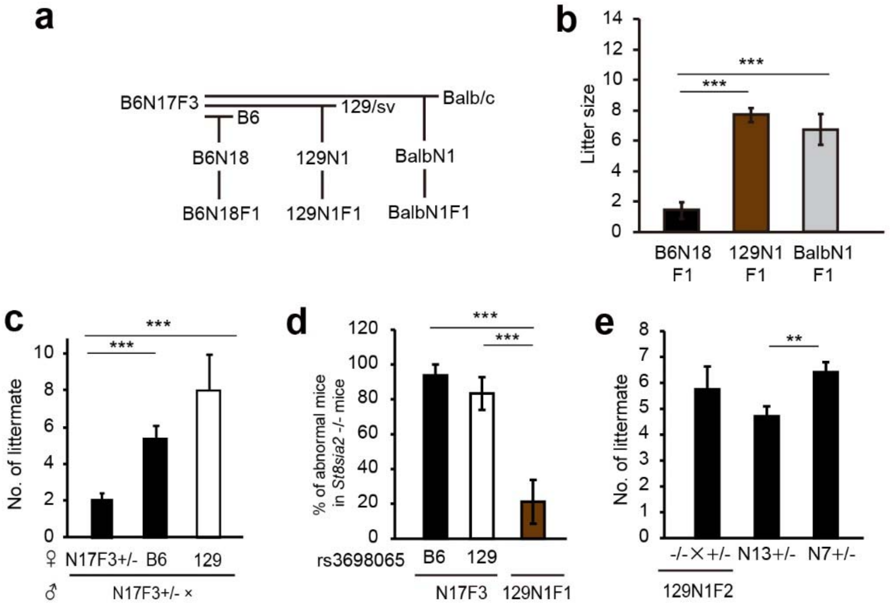

Supplementary figure S3: B6-specific embryonic lethality in *St8sia2* -/- mice.

a, Breeding scheme for backcrossing N17F3+/- with 129 or Balb/c. **b**, Litter sizes from intercrosses of +/- with each genetic background. Mean ± SEM, n = 4-9. ***P < 0.001 (One-way ANOVA, Fisher's least significant difference (LSD) test, P < 0.001). **c**, Litter sizes produced by crosses between male N17 *St8sia2* +/- mice and female N17 +/-, B6, or 129 mice. Mean ± SEM, n = 8-12. ***P < 0.001 (One-way ANOVA, LSD test, P < 0.001). **d**, Ratio of animals showing abnormal phenotypes in null mice of each generation. Mean ± SEM, n = 4-9. ***P < 0.001 (One-way ANOVA, LSD test, P < 0.001). **e**, Litter sizes produced by crosses between 129N1F2 +/- mice and -/- were recovered to a level in early (N7) and middle (N13) stages of backcrossing. **P < 0.01(One-way ANOVA, LSD test, P < 0.05).

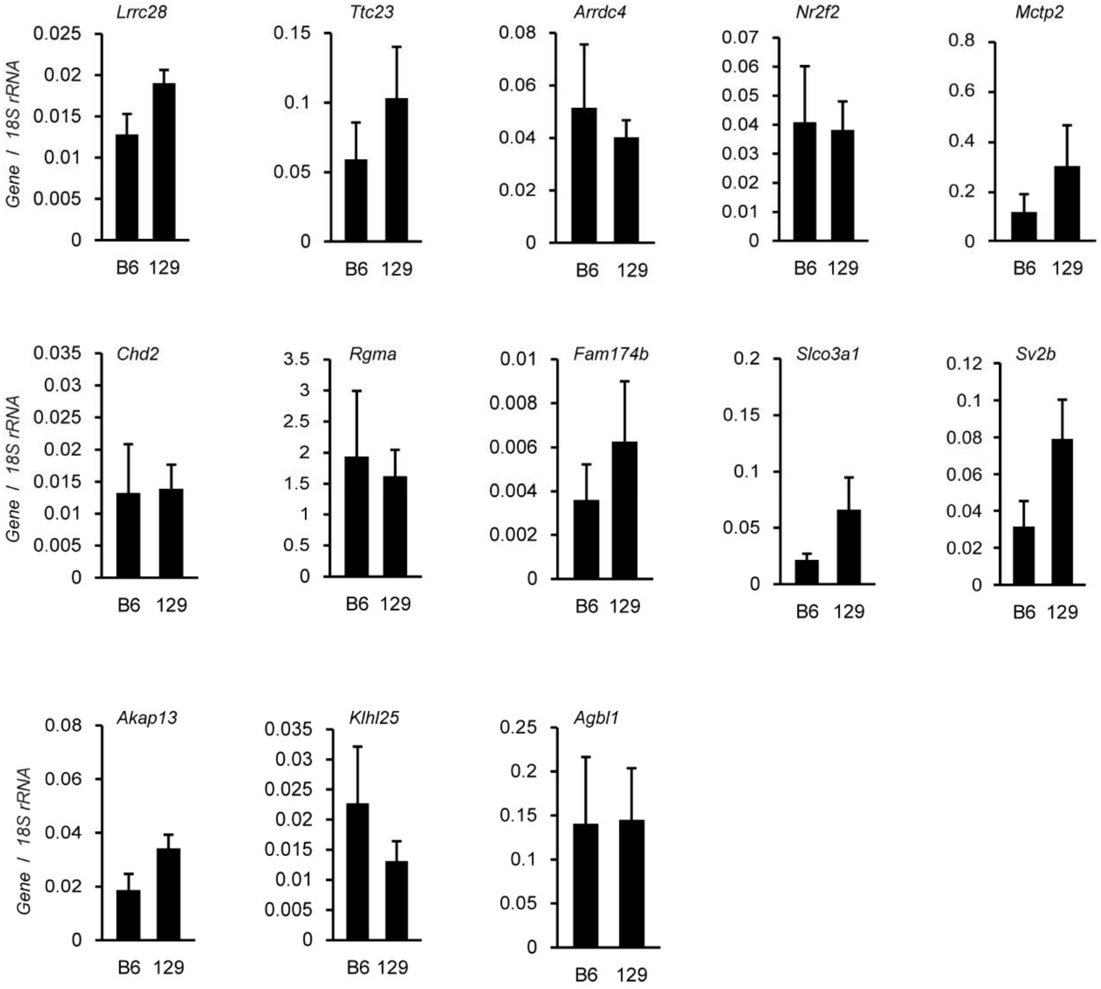

Supplementary figure S4: Gene expression in embryonic heads of *St8sia2* -/- mice on B6 and 129 backgrounds.

Expression profile of all flanking genes except *Synm*, *Igf1r*, and *Pgpep1l* between D7Mit297 and D7Mit30 in the embryonic head (E10.5). The amounts of each mRNA were normalized relative to 18S rRNA levels. No significant changes were observed ($P > 0.05$, *t*-test). Mean ± SEM, n = 4.

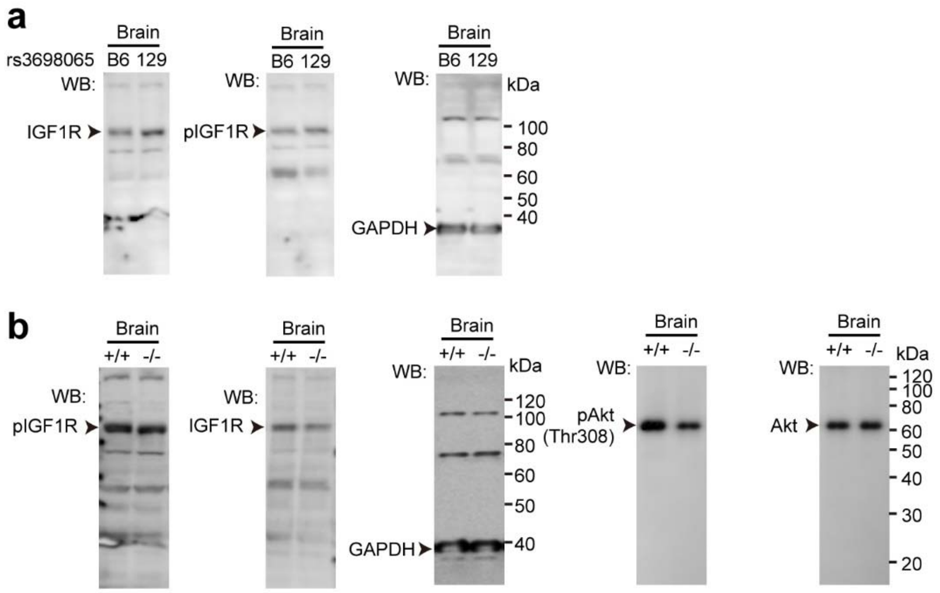

Supplementary figure S5: Full-length blots of IGF1R and pIGF1R in Figure 4

a, IGF1R expression and phosphorylation in embryonic head at E10.5 on B6 and 129 backgrounds at rs3698065. The arrowheads indicate bands showed in Figure 4. **b**, Effect of *St8sia2* deletion on IGF1R expression and phosphorylation of IGF1R and Akt. Left spots in pIGF1R blot image are signals of molecular markers. The arrowheads indicate bands showed in Figure 4.

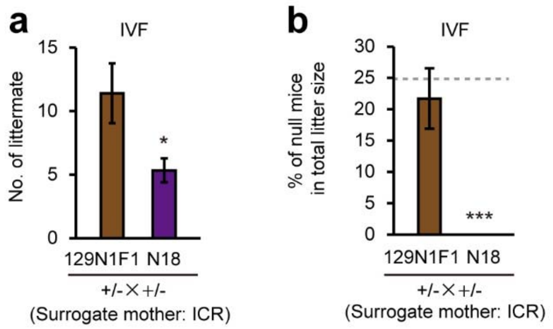

Supplementary figure S6: Maternal effect on embryonic lethality in *St8sia2* -/- mice. **a,b**, Litter size (**a**) and ratios of null mice (**b**) when in vitro fertilization (IVF) embryos in heterozygous intercrossing of 129N1F1 and N18 mice transferred wild-type ICR females. Embryonic lethality is not influenced by their maternal effect. Mean ± SEM, n = 5-9. *P < 0.05, *t*-test.

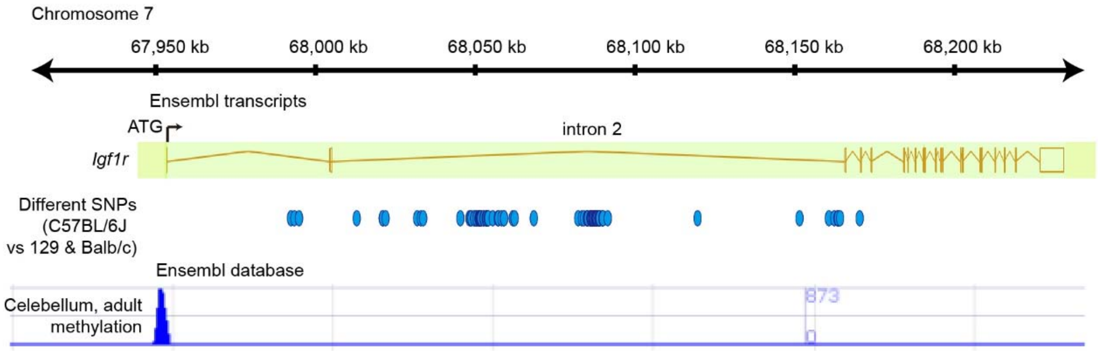

Supplementary figure S7: SNP analysis and hypermethylated site in *Igf1r*.

SNP maps generated from the Mouse Genomes Project - Query SNPs (Wellcome Trust Sanger Institute; https://www.sanger.ac.uk/sanger/Mouse_SnpViewer/rel-1303). SNP maps cover the region surrounding the *Igf1r* gene (coding region ±10 kbp, green belt [top]) in 3 strains of mice. *Igf1r* of B6 is the reference sequence when compared with 129 and Balb/c strains of mice. Light blue ellipses (middle) differences between SNPs of 129 and Balb/c and B6. Many SNPs were observed in intron 2, but a few SNPs were located in other introns. Although high methylation of the *Igf1r* promoter in the cerebellum of adult mice (ESEMBL database) is known (down), no different SNPs were found in the *Igf1r* promoter.

ID	PCR product size (bp)		Forward (5'→3')	Reverse (5'→3')	MGI position (cM)
	B6	129			
D7Mit178	203	165	ACCTCTGATTTCAGAACCCTTG	TAGAGAGCCACTAGCATATCATAACC	0.5
D7Mit22	223	199	AGCCCCTTCTTCTCCAAGAG	TGAAGGAGTAGTCAACAGAGTAAATGC	8
D7Mit156	146	127	CCCTCACCCTCTTTGTCAAA	TCCTTAGGGAAGGGAGTAGACC	16
D7Mit121	145	167	ACCTGCATCTGTGCACATGT	ATTGGAGTTTTATTATACAAGTGGTTG	28.1
D7Mit297	146	161	CCCACAGAGGGCATGAAC	CTGAGGGATTACCTAGATCAATTAGG	35.28
D7Mit30	234	245	CCAGGCTATTCTGTGATGTTTG	GGATGACTCCTAAGGAATGGC	37
D7Mit31	240	232	TTCAAACCATCCAGTAAGTCCA	TTGGTGAACTGCTTCAATGC	44
D7Mit44	305	313	TTCTGGCCTCTGTGAAGTAGTG	GTGAAACCATGGTGCAGATG	49.8
D7Mit281	112	139	TTCCTCTACCTCCTGAGCCA	GCCACAAGGAAGACACCATT	52.4
D7Mit40	203	220	GTCAACAGTCAGGAAAGCTGG	CAGATGCTTGTATTTGCAAAGC	53
D7Mit104	135	154	CACCAAACAGTGTAATAGACATGC	CCAGGGCTTTCATGAGAAAG	63.5
D7Nds4	169	162	GTGACAATACATTCCTGCTGT	CTCAGATCTTATCTCTAGCAC	72.4
D7Mit297	146	161	CCCACAGAGGGCATGAAC	CTGAGGGATTACCTAGATCAATTAGG	35.28

Supplementary table S1: Primer sequences used in microsatellite analysis, Related to Figure 2

Forward (5'→3')		Reverse (5'→3')	Chr Position (bp)
for B6	for 129		
GACTGAGGGGTGATTTCTGAC	GACTGAGGGGTGATTTCTGAT	TTATCAAGTCATCTGAACCA	66128836
GG GCTCATGTATCCGAAGAAGAG	GG GCTCATGTATCCGAAGAAGAA	TGGCACAGTCATCTACCAGG	66278500
GGATGCTGGACACTGGAATCA	GGATGCTGGACACTGGAATCG	TTACAATACTTTAAAACTGA	66433568
ACACTTCCTGAGCGTTCCGTT	ACACTTCCTGAGCGTTCCGTC	CTCCATCCCTGAGACTTTCA	67444261
AGGGTGGAACTCACAGTTGGG	AGGGTGGAACTCACAGTTGGA	GTCAACAGCAGATCTCGTCC	67447250
TGGTGGGTACCACCCATGAAC	TGGTGGGTACCACCCATGAAG	AAGCCTAAGAGGCTTCCTGC	67460289
AAAGCAATGCCTGCAGCCCATCA	AAAGCAATGCCTGCAGCCCATCG	TGAGTCAGAGGAAGTAGACC	67467627
TATTCGAGGACTCCAAGCACG	TATTCGAGGACTCCAAGCACA	AGGCACTAAGATACTTCACTG	67651602
ATGCTCTGCTGCCAGTGCGCC	ATGCTCTGCTGCCAGTGCGCT	ATATTGGCAAATGGAGATGCA	67760472
GAAAGCCATTTCCTATGCGCC	GAAAGCCATTTCCTATGCGCT	AATCTTCAACTTGCTGTATT	68116674
GGGATTCTGCTGCCTTGTTCG	GGGATTCTGCTGCCTTGTTCA	ACACCATGGTACTTACTGCC	68385045
CTAACTTTCACATTGCCACTA	CTAACTTTCACATTGCCACTG	TTGGTTAAGTTTTACTGCAG	69249798
GGAAGCATCTCCAGGCTTACC	GGAAGCATCTCCAGGCTTACA	CTACATCCTCTTTAGCGTGGTC	73188704
CTACATTTGGCTGGCTTGTTA	CTACATTTGGCTGGCTTGTTC	ATGTGTTTGCCCCAAATGAG	73327985
GGCACACTCCGGTTCGGCACG	GGCACACTCCGGTTCGGCACA	TACTGTACATTTGCTGAAAT	74275366
TGCAGCTTTGCTCCCACACTT	TGCAGCTTTGCTCCCACACTC	TGTAAGATAGTCAATCACTA	76415155

Supplementary table S2: Primer sequences used in SNP analysis, Related to Figure 2

Gene	Forward (5'→3')	Reverse (5'→3')
Lrrc28	ACCTCCAGAGGTTGGCGATTTG	AGAGAAAGGCACAGGTGAAGCC
Ttc23	AGCGTACGCGAAGCTAAAGAAGTG	TCACCGATTGCCTTGACTTCCC
Synm	AACAGCGTGGGTCTTGAATGGC	AGGCTACATGGCATTGACACAGC
Igf1r	TGGGCAATGGAGTGCTGTATGC	CCATTCATCAGGCACGTACACATC
Pgpep1l	TCTCCAGCTGTCTGTGCCTTAC	CACACGGGCTGTTTATTGTCTTGG
Arrdc4	TCGCTTTCAGCTTCCGTCTGAAC	ACGCTCTGATCTGGAACTTGGG
Nr2f2	TTCCAAGAGCAAGTGGAGAAGCTC	TCAGACAGACCACAGGCATCTGAG
Mctp2	CTGGATCTGCACAAACCTTCCC	TCGTCATTGCCGTGAGTCTTGTC
Chd2	GCCGTATGAGCAGTACAACAGC	TGGAGTCATGATGGTGCCTGTC
Rgma	TCACAGACCACTTCCAGACATGC	TCTTGAAGATGATGGTGAGCTTGC
Fam174b	TACACCGAACCCTGTTCTCTGC	ATGCTAGGCTCCAGTCCAAAGG
Slco3a1	TCCTGTTTCCAGCAACTGAGAGTG	AGGACGATGCAGGTGAACACAG
Sv2b	TGGAGCATCATTCCACACTATGGC	ACTTCAGAGCCACCATGGACAC
Akap13	TAGGAAATGGAGCTGCCACACC	ACCAGCTTTCTCGGTTGATCGC
Klhl25	ACGGTGAGGATCAGGTTGGTACAC	AGGGTGTGAAGCCTTGTGGAAG
Agbl1	GCCATCCAAGTGCGTGAGTTTG	GGAGCTATTCACGTCGGCATTG
18S	GCACAGTGTTTGTAGAGCCTG	GCCCTGGAACTTATTGATCGGG

Supplementary table S3: Primer sequences used in qPCR, Related to Figure 3 and Supplementary figure S4.
